# Successful endoscopic sclerotherapy with bile duct stenting for a vascular malformation neighboring the duodenal papilla in blue rubber bleb nevus syndrome

**DOI:** 10.1002/deo2.113

**Published:** 2022-04-10

**Authors:** Shingo Unoura, Yosuke Toya, Satoshi Kasugai, Tomo Kumei, Masanao Yamazato, Yutaka Sasaki, Makoto Eizuka, Tomofumi Oizumi, Toshifumi Morishita, Seiya Tagane, Takeshi Shiohata, Shunichi Yanai, Manami Akasaka, Takayuki Matsumoto

**Affiliations:** ^1^ Department of Internal Medicine Division of Gastroenterology School of Medicine Iwate Medical University Iwate Japan; ^2^ Department of Pediatrics School of Medicine Iwate Medical University Iwate Japan

**Keywords:** blue rubber bleb nevus syndrome, duodenal papilla, endoscopic sclerotherapy, polidocanol, vascular malformation

## Abstract

A 14‐year‐old girl, who had been diagnosed with blue rubber bleb nevus syndrome, was referred to our hospital because of iron deficiency anemia. Esophagogastroduodenoscopy revealed a dark and red‐colored vascular malformation occurring just above the duodenal papilla. Because the lesion was regarded as the cause of the anemia, we performed polidocanol injection therapy with bile duct stenting. Since esophagogastroduodenoscopy performed a month later revealed a scarred ulcer, the bile duct stent was removed. She has been under observation as an outpatient without any symptoms.

## INTRODUCTION

Blue rubber bleb nevus syndrome (BRBNS) is a rare systemic disease with multiple vascular malformations (VMs) of the skin, gastrointestinal tract, and other organs.^1^ The skin lesions in BRBNS are often first noticed at birth or in the neonatal period, while those lesions rarely need interventions. In contrast, VM of the gastrointestinal tract can cause acute or chronic bleeding with consequent anemia.^2^

VMs of the gastrointestinal tract occur mostly in the small intestine and distal colon.^3^ To date, there have been some descriptions of small intestinal VMs in BRBNS, which were treated by endoscopic interventions.^2^, ^4^, ^5^, ^6^, ^7^, ^8^, ^9^ However, there are no reports of BRBNS with endoscopic treatment for VM near the duodenal papilla. Herein, we report a case of successful endoscopic sclerotherapy with bile duct stenting for a VM occurring just above the duodenal papilla in BRBNS.

## CASE REPORT

A 14‐year‐old girl was referred to our hospital for a detailed examination of iron deficiency anemia. Her hemoglobin level was 3.5 g/dl at the time of the first visit. She was diagnosed with BRBNS at the age of 10 years due to the VMs in the skin, the cerebellum, the iris, and the duodenum. She also had a prior history of endoscopic sclerotherapy for duodenal VM. The details for the treatment at that time have been reported elsewhere.^1^ Her family history was unremarkable.

**FIGURE 1:**
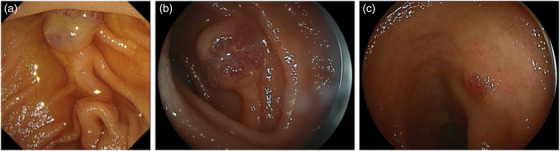
Endoscopic images of the patient. (a) A dark and red‐colored vascular malformation was found just above the duodenal papilla. (b,c) Double‐balloon endoscopy revealed a vascular malformation in the deep portion of the (b) jejunum and (c) ileum (c).

Esophagogastroduodenoscopy revealed a dark and red‐colored VM occurring just above the duodenal papilla (Figure 1a). Capsule endoscopy revealed blood in the upper jejunum and the ileum. Double‐balloon endoscopy revealed vascular malformations in the deep jejunum and ileum (Figure 1b,c). We first treated the VM in the jejunum and ileum by polidocanol injection therapy. Since the capsule endoscopy showed blood in the upper jejunum, we presumed that the duodenal lesion neighboring the papilla was also considered to be the cause of the anemia, and we decided to treat the lesion by endoscopy.

**FIGURE 2:**
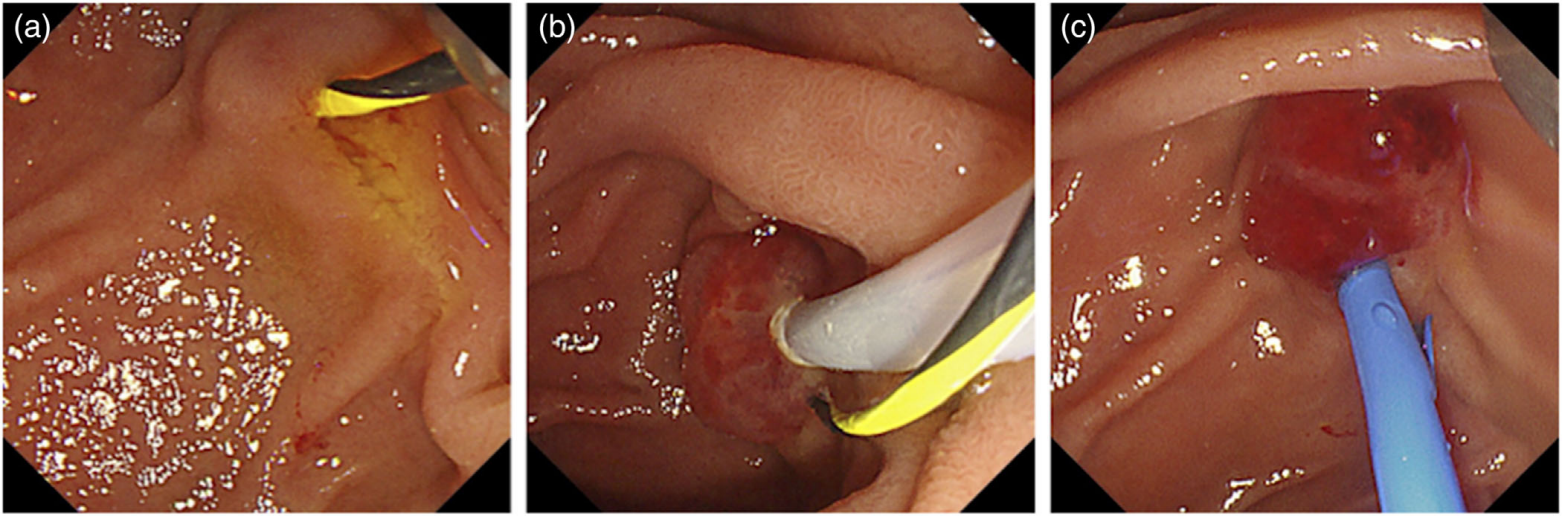
Endoscopic images of polidocanol injection therapy with bile duct stenting. (a) The guidewire was placed in the bile duct. (b) We injected 1.5 ml polidocanol into the center of the lesion. (c) After injection, a bile duct stent was placed

Magnetic resonance cholangiopancreatography showed neither dilatation nor stenosis of the bile duct, while the main pancreatic duct was thin under magnetic resonance cholangiopancreatography. To lessen the risk of cholangitis and to avoid iatrogenic damages in the pancreatic duct, we planned to perform polidocanol injection therapy under the bile duct stenting without pancreatic cannulation. Moreover, because the patient was young, we refrained from endoscopic sphincterotomy to preserve the function of the Oddi sphincter. After the guidewire was placed in the bile duct (Figure 2a), we punctured the center of the lesion and confirmed negative blood backflow. Consequently, we injected 1.5 ml polidocanol in total (Figure 2b). Subsequently, a bile duct stent (7‐Fr, 7 cm, Flexima; Boston Scientific, Massachusetts, USA) was placed (Figure 2c; Supplementary video).

**FIGURE 3:**
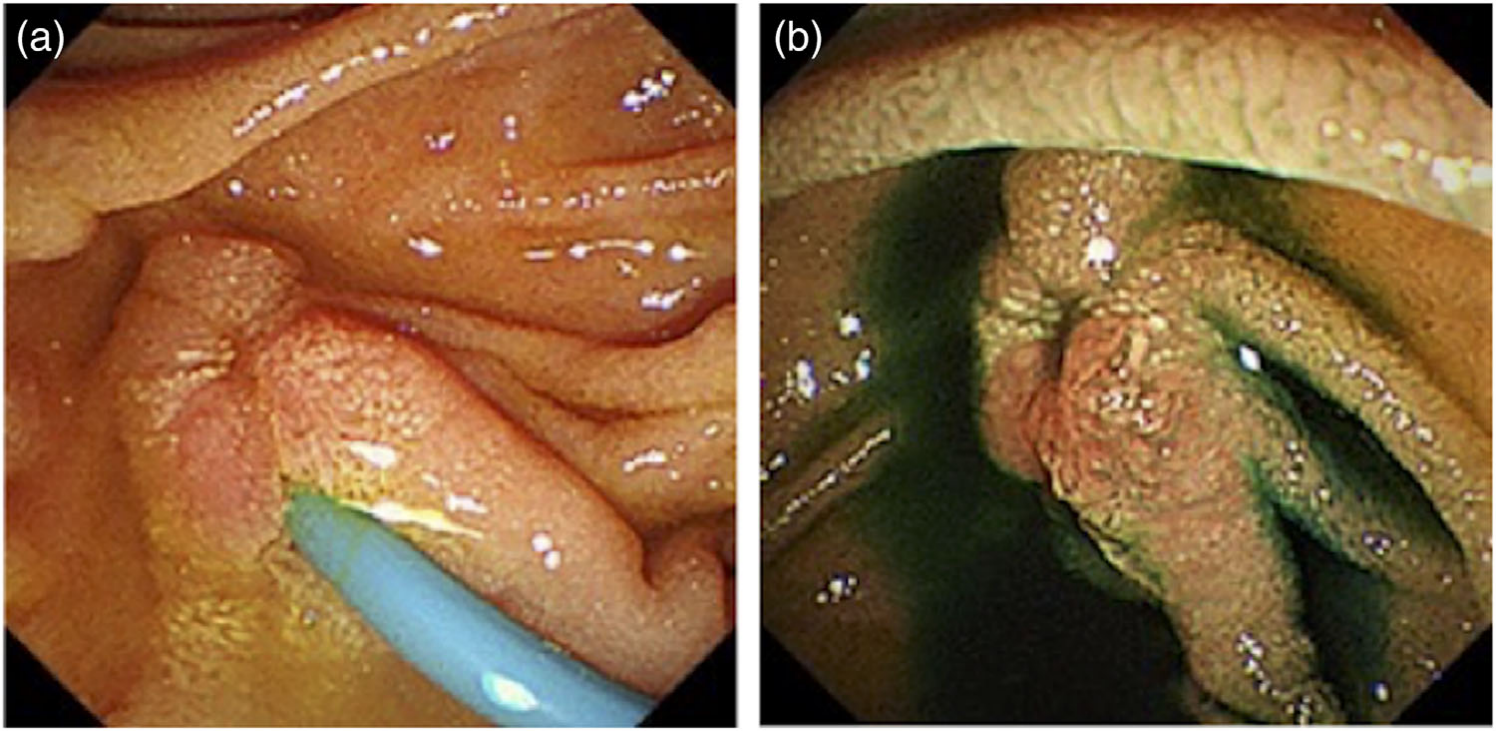
Endoscopic images of after polidocanol injection therapy. (a) One month after treatment, esophagogastroduodenoscopy revealed a scarred ulcer near the ampulla. (b) The bile duct stent was removed

The patient complained of mild abdominal pain for 2 days after the treatment. Serum amylase was slightly elevated to 396 U/L on the day after treatment, but it returned to the normal level on the second day. During the course of the hospitalization, white blood cell count and C‐reactive protein were not elevated. It was thus presumed that her abdominal pain was due to mild pancreatitis. Oral intake was started 3 days after the treatment. The patient was discharged 6 days thereafter. Because esophagogastroduodenoscopy performed a month later revealed a scarred ulcer near the ampulla, we removed the bile duct stent (Figure 3a, b). She has been under observation as an outpatient without any symptoms.

## DISCUSSION

BRBNS is a rare disease characterized by systemic VMs. For VMs in the gastrointestinal tract, surgery, endoscopic treatment or medication can be applied, according to the location, size, and the number of lesions. However, in recent years, endoscopic treatments, especially sclerotherapy, have been increasingly reported due to their less invasiveness and simplicity.

An online search in PubMed of reports published during a period 2015–2021 identified seven cases of endoscopic treatment for duodenal and small intestinal VMs in BRBNS (Table 1).^2^, ^4^, ^5^, ^6^, ^7^, ^8^, ^9^ Of the cases and our present case, five patients were young females, and all the patients presented with symptoms of gastrointestinal bleeding. As for the location of the lesions, the small intestine was the more frequent site than the duodenum. Five of the eight patients were treated with sclerotherapy, two with endoscopic mucosal resection, and the remaining patient with Endoloop ligation. Our case is the first report of sclerotherapy for a VM near the duodenal papilla.

**TABLE 1:** Cases of endoscopic treatment for venous malformation in the duodenum and small intestine in blue rubber bleb nevus syndrome

**Reference**	**Gender**	**Age**	**Symptom**	**Location**	**Treatment**
Ning et al. [2]	M	10	Melena and anemia	Duodenum, jejunum, and ileum	Sclerotherapy (lauromacrogol)
Jin et al. [4]	M	21	Melena and anemia	Ileum	Endoloop ligatation
Wang et al. [5]	F	24	Melena and anemia	Jejunum and ileum	Sclerotherapy (lauromacrogol)
Rubio‐Mateos et al. [6]	F	21	Anemia	Jejunum	Endoscopic mucosal resection
Li et al. [7]	M	13	Melena, dizziness, and fatigue	Jejunum and ileum	Sclerotherapy (lauromacrogol)
Moghadam et al. [8]	F	20	Melena and anemia	Jejunum	Endoscopic mucosal resection
Marakhouski et al. [9]	F	4	Melena and anemia	Small intestine	Sclerotherapy (aethoxysklerol)
Present case	F	14	Anemia	Duodenum, jejunum, and ileum	Sclerotherapy (polidocanol)

Abbreviation: BRBNS, blue rubber bleb nevus syndrome.

Polidocanol has been used for sclerotherapy of vascular lesions in the gastrointestinal tract. Recently, Igawa et al. reported that the optimal dose of polidocanol injection for small‐bowel hemangioma is 0.2 ml per 1 mm of the lesion diameter.^10^ On the bases of this report, we injected 1.5 ml of polidocanol. Although mild abdominal pain was observed after the treatment, there were no serious complications during the clinical course. We believe that the recommended dosage seems to be appropriate, but further validation in a larger number of patients is warranted.

Before treatment, we discussed whether pancreatic duct stenting should be performed. However, because the pancreatic duct depicted by magnetic resonance cholangiopancreatography was very thin, we considered that the stent itself might pose a risk of pancreatitis. Fortunately, the patient did not develop severe pancreatitis after the treatment. However, the indication of pancreatic ductal stenting should be discussed in each case, and a practical and optimal management strategy of VMs at or around the duodenal ampulla should be established.

In conclusion, our experience suggests that polidocanol injection therapy is effective for the lesion near the duodenal papilla in patients with BRBNS. The necessity of bile and pancreatic stenting needs to be elucidated further.

## CONFLICT OF INTEREST

Author Takayuki Matsumoto is the responsible and executive JGES member for DEN Open. The other authors declare no conflict of interest.

## FUNDING INFORMATION

The authors declare no funding for this article.

## Supporting information




**Supplement Video 1**. Video image of polidocanol injection therapy. First, the guidewire was placed in the bile duct. Second, we punctured the center of the lesion and injected 1.5 ml polidocanol. Finally, a bile duct stent was placed.

## References

[1] Kumei T , Toya Y , Shiohata T *et al*. Endoscopic injection sclerotherapy for duodenal vascular malformation in blue rubber bleb nevus syndrome. J Gastroenterol Hepatol 2019; 34: 963. 30638279 10.1111/jgh.14590

[2] Ning S , Zhang Y , Zu Z , Mao X , Mao G . Enteroscopic sclerotherapy in blue rubber bleb nevus syndrome. Pak J Med Sci 2015; 31: 226–8. 25878650 10.12669/pjms.311.5858 PMC4386193

[3] Gallo SH , McClave SA . Blue rubber bleb nevus syndrome: Gastrointestinal involvement and its endoscopic presentation. Gastrointest Endosc 1992; 38: 72–6. 1612387 10.1016/s0016-5107(92)70339-3

[4] Jin J , Pan J , Zhu L . Therapy for hemangiomas of blue rubber bleb nevus syndrome in the small intestine with single balloon endoscopy. Dig Endosc 2015; 27: 780. 26250645 10.1111/den.12522

[5] Wang Z , Yang X , Wu L *et al*. Blue rubber bleb nevus syndrome: Treatment of lesions in the small intestine with repeated injection of lauromacrogol. Gastrointest Endosc 2015; 81: 1274–5. 25442079 10.1016/j.gie.2014.08.013

[6] Rubio‐Mateos JM , Tojo‐González R , Pérez‐Cuadrado‐Robles E . Endoscopic mucosal resection by double‐balloon enteroscopy can be an alternative in small bowel venous malformations. Dig Endosc 2018; 30: 789. 29947439 10.1111/den.13224

[7] Li A , Chen FX , Li YQ . An Unusual Cause of Recurrent Melena. Gastroenterology 2019; 157: 311–2. 30981790 10.1053/j.gastro.2019.04.009

[8] Dooghaie Moghadam A , Bagheri M , Eslami P *et al*. Blue rubber bleb nevus syndrome because of 12 years of iron deficiency anemia in a patient by double balloon enteroscopy: A case report and review of literature. Middle East J Dig Dis 2021; 13: 153–9. 34712454 10.34172/mejdd.2021.219 PMC8531930

[9] Marakhouski K , Sharafanovich E , Kolbik U *et al*. Endoscopic treatment of blue rubber bleb nevus syndrome in a 4‐year‐old girl with long‐term follow‐up: A case report. World J Gastroint Endosc 2021; 13: 90–6. 10.4253/wjge.v13.i3.90 PMC7958468 33763189

[10] Igawa A , Oka S , Tanaka S , Kunihara S , Nakano M , Chayama K . Polidocanol injection therapy for small‐bowel hemangioma by using double‐balloon endoscopy. Gastrointest Endosc 2016; 84: 163–7. 26907744 10.1016/j.gie.2016.02.021

